# Recent Trends in the Application of Chromatographic Techniques in the Analysis of Luteolin and Its Derivatives

**DOI:** 10.3390/biom9110731

**Published:** 2019-11-12

**Authors:** Aleksandra Maria Juszczak, Marijana Zovko-Končić, Michał Tomczyk

**Affiliations:** 1 Department of Pharmacognosy, Faculty of Pharmacy, Medical University of Białystok, ul. Mickiewicza 2a, 15-230 Białystok, Poland; aleksandra.juszczak@umb.edu.pl; 2 Department of Pharmacognosy, University of Zagreb, Faculty of Pharmacy and Biochemistry, A. Kovačića 1, 10000 Zagreb, Croatia; mzovko@pharma.hr

**Keywords:** luteolin, hyphenated techniques, chromatography

## Abstract

Luteolin is a flavonoid often found in various medicinal plants that exhibits multiple biological effects such as antioxidant, anti-inflammatory and immunomodulatory activity. Commercially available medicinal plants and their preparations containing luteolin are often used in the treatment of hypertension, inflammatory diseases, and even cancer. However, to establish the quality of such preparations, appropriate analytical methods should be used. Therefore, the present paper provides the first comprehensive review of the current analytical methods that were developed and validated for the quantitative determination of luteolin and its *C*- and *O*-derivatives including orientin, isoorientin, luteolin 7-*O*-glucoside and others. It provides a systematic overview of chromatographic analytical techniques including thin layer chromatography (TLC), high performance thin layer chromatography (HPTLC), liquid chromatography (LC), high performance liquid chromatography (HPLC), gas chromatography (GC) and counter-current chromatography (CCC), as well as the conditions used in the determination of luteolin and its derivatives in plant material.

## 1. Introduction

**Figure 1:**
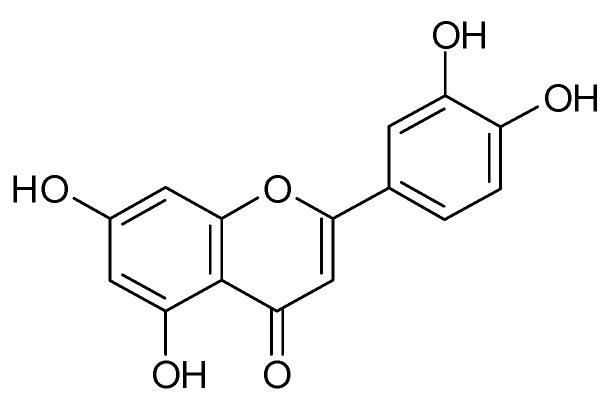
Chemical structure of luteolin.

Luteolin (Figure 1) is a yellow dye commonly found in fresh plants. It is a flavonoid of the flavone type that is distributed widely throughout the plant kingdom. Similar to other derivatives of 2-phenylbenzo-γ-pyrone, its basic skeleton has a characteristic C_6_-C_3_-C_6_ system, containing two benzene rings and a bridge with a C2-C3 double carbon bond and an attached oxygen atom [1,2,3,4]. Structure-activity studies have demonstrated that the pharmacological effects of luteolin and other flavonoids are strongly related to the presence of hydroxyl groups at the C5, C7, C3’ and C4’ carbons as well as to the presence of the double bond in the C2-C3 position [3,5]. The presence of the -OH group at position C3’ distinguishes luteolin from apigenin, and the lack of this group at the C3 carbon is an element that places luteolin in the flavone group [6].

Luteolin exhibits multiple biological effects such as antioxidant, anti-inflammatory and immunomodulatory activity. Plants rich in luteolin are often used in traditional medicine for treatment of various diseases such as hypertension, inflammatory disorders, and even cancer [7].

Because luteolin bears four hydroxyl groups (at the C5, C7, C3’ and C4’ positions), many derivatives of luteolin can be created. Various types of functional groups and/or sugar molecules can be attached to those positions, creating a huge number of different but structurally similar molecules. The most common are methyl derivatives, as well as *C*- and -*O*-glycosides [8,9].

Orientin, an 8-*C*-glucoside derivative of luteolin, displays an array of health-related biological properties, such as antioxidant, anti-ageing, antiviral, antibacterial, anti-inflammatory, vasodilatation, cardioprotective, radiation protective, neuroprotective, antidepressant-like, anti-adipogenesis, and antinociceptive effects. It may be found in different medicinal plants such as *Ocimum sanctum* (holy basil), *Phyllostachys nigra* (bamboo leaves), *Passiflora* sp. (passion flower), *Linum usitatissimum* (flax), *Euterpe oleracea* (Acai palm) and many others [10]. Another luteolin derivative, isoorientin (luteolin-6-*C*-glucoside) acts as an antioxidant, photoprotective [11], skin lightening [12], hepatoprotective [13] and anti-inflammatory agent [14]. *O*-glucosides of luteolin also display biological activities. For example, luteolin 7-*O*-glucoside alleviates skin lesions in murine models of atopic dermatitis [15] and protects cells against apoptosis induced by hypoxia/reoxygenation [16].

An increasing number of herbal preparations on the market contain luteolin and its derivatives, either as single-ingredient products or in mixtures with other phytochemicals, e.g., in form of medicinal plants extracts. To establish the quality of such products, it is important to use appropriate analytical methods. However, there is a lack of quality reviews of the available methods for quantification of luteolin derivatives, and the information on their comparison is lacking. Therefore, the aim of this article is to systematize knowledge and information in the field of chromatographic analytical techniques used for quantification of luteolin and its derivatives. The presented review is the first description of this type and provides a systematic overview of chromatographic analytical techniques including thin layer chromatography (TLC), high performance thin layer chromatography (HPTLC), liquid chromatography (LC), high performance liquid chromatography (HPLC), gas chromatography (GC) and counter-current chromatography (CCC), as well as the conditions used to assess luteolin and its derivatives.

## 2. Chromatographic Techniques for the Analysis of Luteolin Derivatives

Chromatography occupies a leading position among other instrumental methods in the analysis of chemical compounds. As a physicochemical method of separation and analysis of mixtures of chemical compounds, it allows detection and identification as well as quantitative determination of the test substance with high accuracy. The coupling of chromatography with other methods of analysis contributes to a more accurate detection and expansion of analytical capabilities, especially for complex mixtures of organic compounds [2,17,18].

Chromatographic techniques are based on the interaction of the mixture components with the mobile and stationary phases of the chromatographic system. This results in the division of the mixture components between the two phases. In addition, the interaction of the mobile and stationary phases is also important in the separation process [17,18]. According to the aggregation state of the mobile phase, chromatographic techniques are divided into gas, liquid and supercritical chromatography. Another criterion for classification of chromatographic techniques is the type of stationary phase. If the stationary phase is a liquid, the chromatography technique is referred to as partition chromatography. In the case of a solid, the technique is referred to as adsorption chromatography. Another example of the classification of chromatographic methods is their division depending on the chromatographic processing method. This classification allows distinguishing column chromatography and planar techniques, which include TLC and paper chromatography [17,19]. Many different chromatographic techniques are used in the analysis of luteolin derivatives. These include TLC, HPTLC, LC, HPLC, GC and CCC [19].

### 2.1. Thin Layer Chromatography in the Analysis of Luteolin Derivatives

Thin layer chromatography (TLC) is a rather simple but relatively popular method used in the analysis of flavonoids since 1960 [20]. It is a variation of LC that is carried out on a plane and is therefore referred to as planar chromatography. Despite the dynamic development of other chromatography techniques, TLC has not lost its importance in phytochemical analysis [18]. Its values are still recognized as is the basic tool in the qualitative analysis of natural products, and as such, it is still recommended by most modern Pharmacopoeias. In addition, the TLC technique is currently being improved, and the scope of its application is widening, while the results are becoming comparable to those obtained by GC or HPLC [20,21,22]. The stages of chromatographic analysis consist of placing the sample on the stationary phase and developing the chromatogram, followed by its visualization. In the final step, qualitative and/or quantitative determinations of the tested components are made [18,22,23]. General guidelines for flavonoid separation on TLC plates are presented in Table 1.

The main advantage of TLC is that it is a relatively simple and inexpensive technique that allows for rapid qualitative and quantitative analysis of the tested compounds. Samples analyzed with this method usually do not require pre-treatment, such as purification or concentration. In addition, several dozens of samples can be analyzed simultaneously on one plate. A large amount of the diluted sample can be applied to the stationary phase because the solvent evaporates during the application to the plate. Furthermore, due to the evaporation of the solvent phase after the development of the chromatogram, the detection method does not depend on the type of mobile phase used for separation. In TLC, it is possible not only to compare the analyzed components with the standards, but also to differentiate between substances bearing specific functional groups using the appropriate reagents for detection [18].

Thin layer chromatography and column chromatography (CC) are interchangeable techniques that may be combined, which significantly reduces costs and analysis time [22]. To achieve this, the same adsorbents are ideally used for both TLC and CC [18,22,24]. Nevertheless, other solvent systems may also be used. Elution in CC may be carried out in one mobile phase, or its composition may be changed during chromatography (mobile phase gradient), thereby increasing the elution force. In this case, the TLC mobile phase should be changed accordingly [18,24].

The type and quality of the stationary phase greatly affect the separation of mixture components. Thus, the selection of an appropriate adsorbent is very important. However, most TLC analyses are carried out in a normal phase system where hydrophilic (polar) adsorbents are used [18,23,24]. Reversed phase systems with lipophilic (non-polar) stationary phases are common and have little significance in the analysis of flavonoids [20]. Currently, the most commonly used stationary phase for the analysis of flavonoids is silica gel [20]. However, the use polyamide coated chromatography plates, in both normal and reversed phase systems, is not uncommon [24].

Detection of flavonoids on TLC plates is most often conducted under ultraviolet (UV) light at wavelengths of 254 or 366 nm. Luteolin derivatives also display fluorescence, which can be enhanced using the appropriate derivatization reagents, e.g., with the so-called NP/PEG reagent. The most frequent procedure consists of spraying the plate with 1% methanolic diphenylboric acid-β-ethylamino ester (natural product reagents, NP), followed by 5% ethanolic polyethylene glycol 4000 (PEG) solution [20]. A densitometer may also be used for the qualitative analysis of the substance. The analysis is performed by comparing the retardation factor (Rf) and absorption spectrum of the test substance and the standard. Analytes can also be identified by extracting the separated substances from the plate. Then, the analysis is carried out using Fourier transform infrared mass spectrometry (MS), UV spectrometry, Raman spectrometry or other techniques [23]. Even though TLC separation of luteolin derivatives (Table 2) can be performed in different types of stationary phases such as a polyamide phase [26,28], it is most frequently performed on silica gel plates that are often coated with a fluorescent indicator [27,29] (F_254_ plates) for preliminary detection. Such an approach has been used in case of analysis of luteolin 3’-*O*-glucoside, luteolin 6,8-*C*-dihexoside and luteolin 7-*O-*rutinoside in *Phlomis* sp. [27]. However, subsequent analysis with NP/PEG is the standard procedure for TLC analysis of luteolin [26,27,28,29], and it is almost always performed regardless of additional types of detection such as detection of flavonoids in *Ligustrum vulgare* with aniline phthalate [26]. Typically, the mobile phase for luteolin derivative separation consists of a mixture of aprotic organic solvents such as ethyl acetate (EtOAc) [26,28] or acetone (Ace) [29] and H_2_O with a significant amount of formic (FA) and/or acetic acid (AcOH) to avoid tailing of the separated zones [26,27,28,29].

Thin layer chromatography is often used as a complementary method to other chromatographic techniques. For example, analysis of the butanol (BuOH) fraction of the methanol (MeOH) extract from the leaves of the common privet (*Ligustrum vulgare*) conducted by Mučaji et al. [26] allowed isolation of two luteolin derivatives from the plant. TLC was carried out, among other techniques, on polyamide plates, and the optimal mobile phase was found. TLC was used for analysis of individual fractions obtained in column chromatography with or without acid hydrolysis of compounds. MS detection and nuclear magnetic resonance (NMR) spectra were also used [26]. Furthermore, TLC was used together with high performance liquid chromatography combined with mass spectrometry and pulsed amperometric detection (HPLC-PAD-MS) for analysis of luteolin and other phenolic compounds in *Leontopodium alpinum*. In addition to NP/PEG, UV, infrared (IR) and NMR analyses were used for identification of these compounds [29].

Compared to other chromatographic techniques, HPTLC results in reduced time and costs of analysis and provides much greater efficiency of separation. It is suitable even for the analysis of crude extracts similar to TLC, and a relatively small amount of solvent is used to analyze several samples, making this method environmentally friendly [60]. In the analysis of luteolin derivatives (Table 3), HPTLC silica gel 60 is almost exclusively used as the stationary phase [45,47,49,51], while HPTLC NH_2_ plates are rarely used, e.g., for separation of flavonoids in some Lamiaceae species such as *Mentha piperita* [53] and *Thymus* sp. [55]. In addition to NP/PEG (e.g., [52,57]), other detection systems may be employed for visualization of luteolin derivatives, such as bis-diazotized sulfanilamide [53] or aqueous solutions of Al^3+^ ions for flavonoids in *M. piperita* [53], honey [49] or *Thymus* sp. [55]. Similar to TLC, mixtures of organic solvents, H_2_O, and FA are most often used as the mobile phase [44,45,47,48,49,51,52].

High performance thin layer chromatography may also be used as a complimentary method to other chromatographic techniques. Chelyn et al. [44] used the HPTLC technique in the analysis of the ethanol (EtOH) extract of *Clinacanthus nutans* leaves. The analysis revealed the presence of, among others, luteolin 8-*C*-glucoside (orientin) and luteolin 6-*C*-glucoside (isoorientin) in the raw material. Their detection was conducted by comparing R_f_ coefficients using derivatization reagents and 366 nm UV light. In this work, the characteristic fluorescent bands after derivatization provided important clues for the identification of the major flavone present in the samples, while high performance liquid chromatography combined with ultraviolet spectrometry or a diode array detector (HPLC-UV/DAD) technique was employed for the simultaneous detection and quantification of these compounds [44].

### 2.2. High Performance Liquid Chromatography in the Analysis of Luteolin Derivatives

Among the many chromatographic methods, adsorptive chromatography, in which the mobile phase is liquid and the stationary phase is solid, is of the greatest practical importance. For example, this method has a much wider application than GC because it allows the analysis of substances in the form of liquids and soluble solids. Furthermore, it is also suitable for analysis of thermolabile substances because it is usually performed at low temperatures, and such samples are not degraded [61]. Due to the long analysis time, high mobile phase consumption and low efficiency, traditional CC is currently used mainly for preparative purposes. However, improved methods, such as HPLC and especially liquid chromatography combined with mass spectrometry (LC-MS), are increasingly used for analysis of natural compounds including luteolin derivatives [62].

High performance liquid chromatography has been performed since 1960. It is a dynamically developing method with a wide range of uses and has been proven to be very useful in phytochemical analysis. The principle of operation consists of pumping the mobile phase from the tank (or tanks) through the stationary phase-filled column. Eluents are previously filtered and degassed. Some systems are additionally equipped with thermostats that regulate the temperature of the column. If the chromatographic system is properly selected and applied, then the individual components are separated and detected. Strengthened signals are transmitted to the computer, where the obtained data are registered and properly processed [19]. The separation is based on competition of the molecules of the eluent and the substance being analyzed for the space on the adsorbent surface in the column [63,64].

When choosing a column, one should be guided by the size of the sample, time of analysis and expected effect of the separation. Although columns with different diameters are available, those with a diameter of 4.6 mm are by far the most common. However, due to better detection of the components separated in smaller diameter columns, these columns are increasingly being used for separation and analysis [19,63,65]. In addition, improved separation can be achieved using a column filled with smaller particles. In addition to 5 μm particles, which are most common, particles of 3 μm in diameter or even smaller can be used. For example (Table 4), this approach was chosen when separating luteolin 2’’-*O*-feruloylhexosyl-6-*C*-hexoside and luteolin 6-*C*-glucoside from *Arenaria montana* [66,67] and isoorientin in *Achillea millefolium* [68,69], as well as separating luteolin 6-*C*-hexosyl-8-*C*-pentoside, luteolin 2’’-*O*-deoxyhexosyl-6-*C*-glucoside and luteolin 6-*C*-glucoside in *Cymbopogon citratus* [70,71]. Particles less than 2 μm in diameter were used for ultra-performance liquid chromatography (UPLC) analysis of luteolin derivatives and other flavonoids in *Lactuca sativa* [72,73] and date palm (*Phoenix dactylifera*) [74].

Stationary phases with different polarity may be used in HPLC. In the normal phase system, polar column fillings are used, and most often the filling is silica gel. However, silica gel can adsorb water, which leads to the loss of the original separating properties of the column and thus to impaired reproducibility of the obtained results [19,65]. Therefore, the gel is often modified with the aim of enabling better separation of mixture components. This is performed mainly by bonding alkyl chains (or alkyl chains bearing other functional groups) to functional groups on the gel surface. Silica gels optimized in this way are referred to as the associated phase [2,65]. In the so-called reversed phase (RP) system, non-polar associated phases are used. Such systems are especially useful in the analysis of insoluble or poorly water-soluble compounds, as well as in the analysis of polar compounds, provided that a mobile phase with high water content is used. Typically, an octadecylsilane (ODS) phase, composed of 18 carbon atoms (RP-18), is employed. Such fillers can have different properties depending on the silica gel type and/or production method [19,65]. According to the available literature, the analysis of luteolin derivatives (Table 4) was performed exclusively with RP systems, and the RP-18 system was the most frequently used system, as exemplified in the separation of six luteolin derivatives in *Securigera securidaca* [75] or as many as ten derivatives in *Capsicum annuum* [76]. Stationary phases composed of 8 carbon atoms (RP-8) were rarely used. Examples include the separation of flavonoids in *Coriandrum sativum* [77] and *Achillea millefolium* [68,69].

The selection of an appropriate mobile phase is extremely important for chromatographic separation. The type of analyte, mixture composition, stationary phase and detector used should be taken into account. Mixtures of up to three components are most commonly used. In the reverse system, mixtures of MeOH/H_2_O or acetonitrile (ACN)/H_2_O are routinely used. As the amount of organic solvent increases, the retention time for non-polar substances decreases, while the addition of H_2_O extends their retention times. As a rule of thumb, systems containing ACN/H_2_O eluents are more efficient than those containing MeOH/H_2_O [19]. In the normal phase system, however, the base solvent is a non-polar eluent, and its polarity is appropriately modified by the addition of another solvent of higher polarity, e.g., chloroform (CHCl_3_) [65]. According to the available literature, virtually all mobile phases used for analysis of luteolin derivatives consisted either of ACN/H_2_O, e.g., [26,66,67,78] or, less often, MeOH/H_2_O mixtures [72,79,80,81]. Additionally, a modifier such as FA, e.g., [79,82,83,84], AcOH, e.g., [44,78,85,86,87], or trifluoroacetic acid (TFA) [88] was added to avoid peak tailing [89]. Analyses performed without acidic modifiers are rare [90].

In the chromatography process, the elution can be carried out in two ways. The first method is isocratic elution, which involves running with the same composition of mobile phase during the analysis. This means that a constant elution force is maintained throughout the entire separation period. In this case, even a slight change in the composition of the mobile phase may affect the results of the analysis. Isocratic elution is only rarely used for analysis of flavonoids. In the case of luteolin derivatives, this method was used only for analysis of luteolin 7-rhamnosyl- (1-6)-galactoside in *Filago germanica* [91], luteolin 7-rhamnosyl(1-6)galactoside in *Galactites elegans* [90], and various luteolin derivatives in *Apium graveolens* [80]. If the composition of the eluent changes during the division of the mixture, then the gradient elution can be described. The gradient can be linear or specifically programmed. With a change in the composition of the eluent, its elution force increases. For this reason, this method is used particularly for the separation of mixtures composed of substances of different polarity [2,64]. The vast majority of papers describing the separation of luteolin derivatives in plant mixtures use a gradient approach. Examples include the separation of luteolin 8-*C*-glucoside, luteolin 6-*C*-glucoside, luteolin 4’-*O*-glucoside and luteolin 7-*O*-glucoside from *Chrysanthemum trifurcatum* [92], thirteen luteolin derivatives from *Cymbopogon citratus* [70,71] and many others.

By far, the most common method for detection of luteolin derivatives is using diode array detectors (DADs) (also called photodiode array detectors, PDA), which were used in as many as 46 instances (e.g., for the analysis of luteolin derivatives and other flavonoids in *Cymbopogon citratus* [79], *Dianthus versicolor* [88], *Clinacanthus nutans* [44], *Thymus alternans* and others [93]). More often than not, however, DADs were combined with other detectors for additional structure determination or confirmation, with or without prior isolation. Examples of DADs combined with other detection methods for analysis of luteolin derivatives in plants include the use of tandem mass spectrometry (MS/MS) and NMR for *Ligustrum vulgare* [26], diode array detectors combined with electrospray ionization and mass spectrometry (DAD-ESI-MS) for *Phoenix dactylifera* [94] and *Nerium indicum* [95], electrospray ionization combined with tandem mass spectrometry (ESI-MS/MS) for *Achillea moschata* [96], electrospray ionization combined with time of flight mass spectrometry (ESI-TOF-MS) and electrospray ionization combined with ion trap and tandem mass spectrometry (ESI-IT-MS/MS) for *Aloysia citrodora* and many others.

For example, the HPLC-PAD-MS technique was used in the analysis of aerial flowering parts of the Edelweiss alpine region (Leontopodium alpinum). This method allowed the basic separation of almost all components of the L. alpinum extract prepared by exhaustive dichloromethane (DCM) followed by MeOH extraction. In total, 14 compounds have been isolated from the extract, including several luteolin derivatives. The authors used a gradient as the mobile phase as follows: H_2_O:0.9% FA:0.1% AcOH:1.5% BuOH (A) and ACN:30% MeOH:0.9% FA:0.1% AcOH (B) and MeOH (C). The structure of the isolated components was additionally confirmed using NMR spectroscopy [29].

### 2.3. Liquid Chromatography in the Analysis of Luteolin Derivatives

LC combined with tandem mass spectrometry (LC-MS/MS) (Table 5) is especially useful in the analysis of multicomponent mixtures, such as herbal extracts because it does not require a large amount of the sample or previous separation. To further reduce the influence of other factors on the analysis, more advanced techniques, involving the combination of more than one detection method, e.g., LC combined with NMR and MS (LC-NMR-MS), are increasingly used [139].

Research on ethanol extract from *Lophatherum gracile* stems and leaves was performed using LC coupled with MS/MS. Gradient elution was performed using 0.3% FA and MeOH. Analysis of the species revealed the presence of, among others, luteolin 7-*O*-β-D-glucoside, and luteolin 6-*C*-glucoside [140].

Another raw material containing luteolin and its derivatives is the cocoa seed (*Theobroma cacao*). In the analysis of the H_2_O:MeOH extract, the following mobile phase was used: H_2_O:0.1% FA and ACN:0.1% FA. The elution was carried out in a linear gradient, whereas LC combined with electrospray ionization and tandem mass spectrometry (LC-ESI-MS/MS) coupling allowed the identification of the tested compounds [141].

Lin and Harnly performed a water-methanol analysis of the flower extract of *Chrysanthemum morifolium* and distinguished many compounds, including numerous derivatives of luteolin. In this case, the mobile phase was a mixture of 0.1% FA:H_2_O and 0.1% FA:ACN, in varying proportions. The qualitative determination of the analyzed substances was based on a comparison of retention times as well as mass and UV/Vis spectra [142].

### 2.4. Gas Chromatography in the Analysis of Luteolin and Its Derivatives

Gas chromatography (GC) is a technique used to analyze volatile as well as non-volatile compounds after their derivatization. GC is characterized by chromatographic distribution of either a gas mobile phase on a solid adsorbent (gas-solid chromatography) or a liquid on an inert support (gas-liquid chromatography). GC can be hyphenated with various detection techniques such as GC combined with mass spectrometry (GC-MS), GC combined with tandem mass spectrometry (GC-MS/MS) or GC combined with time of flight mass spectrometry (GC-TOF-MS), thus greatly increasing the versatility, sensitivity and accuracy of the method [164,165]. The analyzed substances should be thermally stable, and their boiling (or sublimation) temperature should not exceed 350-400°C. To achieve this, non-volatile substances are often derivatived. Polar functional groups are transformed into their less polar counterparts, thus increasing the volatility of the prepared compounds. The most common examples include substitution with a trimethylsilyl (TM) group, organic radicals or compounds such as trimethylchlorosilane (TMCS), hexamethyldisilazane (HMDS) or *N*,*O*-bis(trimethylsilyl)-trifluoroacetamide (BSTFA). Such an approach greatly increases the number of possible analytes [2,17]. For example, phenolic groups of flavonoids are often transformed into their less polar trimethylsilyl counterparts allowing for rapid and effective separation of complex mixtures [166].

In adsorption GC, a gas that is chemically inert to the stationary phase as well as the components being analyzed is used as the mobile phase. Most often hydrogen, nitrogen or argon is used. Helium is being used less often due to its higher cost than other gases and the implementation of the principles of chemical safety. The mobile phase must be properly selected for compatibility with the detector used. However, the carrier gas itself does not have a significant influence on the separation effects of the analyzed mixtures [17]. In the process of separation, the method of application of the sample to the chromatography column is very important. The sample should always have as small of a volume and the shortest dosing time as possible. This ensures better separation and narrower bands [17,164].

Gas chromatography uses open-ended columns, i.e., capillary columns and packed columns. Open-ended (OT-open tubular) columns are characterized by much higher efficiency than packed columns; therefore, they are chosen much more often [164]. Capillary columns are particularly useful in the separation of substances with significantly different boiling points. Ideally, the column should have a similar polarity to the analyzed components. However, due to the higher efficiency and greater durability of stationary phases with low polarity, so capillary columns are recommended for chromatographic analyses [17,164].

Another factor that influences the efficiency of the separation of the analyzed sample components, as well as the time of analysis, is temperature. The separation temperature should be selected depending on the stationary phase used and the boiling point of the analytes [17,164].

The analysis of luteolin derivatives has been carried out in accordance with the general procedure used for analysis of other flavonoids (Table 6) [165]. MS was used for detection of volatile derivatives of luteolin, while the use of a flame ionization detector (FID) was described only in two papers [167,168]. Most commonly, helium was used as a carrier gas (e.g., [169,170,171]), but the use of nitrogen was also recorded [167,168]. The derivatization of luteolin, which is necessary for chromatographic separation, was mostly achieved with BSTFA/TCMS [170,171,172]. For the analysis of lipophilic luteolin derivatives, such as 7,3’,4’-trimethyl-luteolin in *Arnica alpina,* no derivatization was necessary [168].

### 2.5. Counter-Current Chromatography in the Analysis of Luteolin Derivatives

Counter-current chromatography (CCC) is a variation of liquid chromatography in which both the stationary and mobile phases are liquid. The separation of the constituents of the mixture is carried out in a system of immiscible liquids that are in equilibrium with each other. The method is simple and rapid, offering the possibility of introducing the raw sample to the column without need for previous clean-up [173]. Counter-current chromatography is mainly used for purification of natural compounds, while its use as an analytical technique is far less common. In CCC, various dividing techniques can be used, thus distinguishing centrifugal partition chromatography (CPC) and rapid- or high-speed CCC (HSCCC), often referred to as hydrodynamic chromatography [174,175]. High-speed CCC is particularly often encountered in studies involving the separation of flavonoids [176]. Table 7 presents the conditions for the separation of mixtures containing luteolin and its derivatives using rapid counter-current chromatography. Separation of luteolin derivatives from mixtures of different phytochemicals is usually carried out with mixtures of EtOAc with one of alcohol (BuOH, EtOH or AcOH) and H_2_O. Upon separation, structure determination by NMR is often necessary [173,177]. However, instead of NMR, high resolution mass spectrometry (HRMS) can also be employed for structure confirmation, as evidenced by the use of this technique for differentiation of various luteolin derivatives in *Lippia origanoides* [178].

## 3. Conclusions

The presented comparison of chromatographic methods currently used to determine luteolin and its derivatives provides a systematic summary of the available knowledge. Without a doubt, chromatographic analysis may be successfully employed as an efficient method for both qualitative assessment (fingerprinting) and quantitative determination of luteolin derivatives. In tedious and time-consuming determinations of multi-ingredient plant extracts, including those that contain the most prevalent luteolin derivatives, combinations of large-scale chromatographic techniques with serially aligned detection modalities such as LC-MS/MS or LC/NMR/MS were found to be particularly useful. Such beneficial coupling of chromatography with other analytic techniques expands analytical capabilities while additionally improving the accuracy, sensitivity, and precision of assays. Despite the dominant position of LC in the analysis of natural compounds and the dynamic development of novel chromatographic methods, the TLC/HPTLC has not lost its important place in the phytochemical analysis of luteolin derivatives. The technique is relatively simple and inexpensive while facilitating rapid qualitative and quantitative analysis of test compounds. In addition, it facilitates large quantities of diluted samples being deposited in the stationary phase, allowing for a wider choice of mobile phase carriers. The technique is subject to continuous improvements and its range of applicability is expanding.

Effective chromatographic analysis in the determination of luteolin derivatives requires appropriately selected chromatographic separation conditions. The appropriate choice of stationary phase sorbent may significantly improve test conditions. Most TLC analyses of luteolin derivatives are carried out in normal phase systems featuring a polar (hydrophilic) sorbent phase. In HPLC, separation conditions can be chosen more arbitrarily as the technique allows for the use of stationary phases of varying polarity which is particularly advantageous in the analysis of compounds that are either insoluble or poorly soluble in water. In order to additionally improve the sensitivity of HPLC analyses, one should focus on the column parameters responsible for appropriate separation. Quite often, the fairly successful analyses are used as starting points for further modifications, including the development of preparative-scale analyses.

Of all chromatographic techniques, GC is the least applicable in the analysis of plant extracts, including those that contain luteolin derivatives. Despite its high sensitivity and efficiency when coupled with various detection techniques (MS, MS/MS, TOF-MS), the chromatographic separation process involves high temperatures and derivatization of analytes. Due to this important aspect, GC-MS is less frequently used in the analysis of polyphenolic compounds.

The complexity of plant matrices is unquestionably a significant problem in the chromatographic analysis of plant extracts. It has a negative impact on the efficacy of analyses and prevents complete identification of all components of the plant extract. However, chromatography remains the primary and most effective analytical technique available in the current state of the art for the determination of compounds of natural origin.

## Figures and Tables

**Table 1:** Recommended combinations of solvents / adsorbents for identification. of different flavonoid types by thin layer chromatography (TLC).

Flavonoid Type	Adsorbent Type/Mobile Phase		
	Cellulose	Polyamide	Silica gel
Polar flavonoid aglycones, e.g., flavones	BuOH:AcOH:H_2_O (3:1:1 *v/v/v*) ^a^ CHCl_3_:AcOH:H_2_O (30:15:2 *v/v/v*) ^b^	MeOH:AcOH:H_2_O (18:1:1 *v/v/v*)	To:Py:FA (36:9:5 *v/v/v*)
Non-polar flavonoid aglycones, e.g., methylated flavones	10–30% AcOH	---	CHCl_3_:MEOH (15:1 to 3:1 *v/v*)
Flavonoid glycosides	BuOH:AcOH:H_2_O (3:1:1 *v/v/v*) ^a^ BuOH:AcOH:H_2_O (4:1:5 *v/v/v*) ^a^	H_2_O:MeOH:MEK:methyl acetylacetone (13:3:3:1 *v/v/v/v*)	EtOAc:Py:H_2_O:MeOH (80:20:10:5 *v/v/v/v*) (especially flavone C-glycosides)

Abbreviations: AcOH, acetic acid; BuOH, butanol; CHCl3, chloroform; EtOAc, ethyl acetate; FA, formic acid; MeOH, methanol; MEK, methyl ethyl ketone; Py, pyridine; To, toluene. ^a^ The mobile phase is thoroughly mixed in the separating funnel and the upper phase is used. ^b^ The mobile phase is thoroughly mixed in the separating funnel and the excess water is discarded.

**Table 2:** Thin layer chromatography in the analysis of luteolin derivatives.

Luteolin Derivative	Stationary Phase	Mobile Phase	Detection	Analyzed Species	Ref.
Luteolin	silica gel 60 F_254_	Hx:EtOAc:AcOH (31:14:5 *v/v/v*); To:DI:AcOH (90:25:4 *v/v/v*)	FBS; UV, 254, 366 nm	*Artemisia annua*	[25]
	silica gel 60 RP-18 F_254_S	FA:H_2_O:MeOH (5.5:47.25:47.25 *v/v/v*)			
Luteolin 7-rutoside	silica gel	EtOAc:FA:AcOH:H_2_O (100:11:11:23 *v/v/v/v*)	NP/PEG, aniline phthalate; UV, 254,366 nm MS, NMR	*Ligustrum vulgare*	[26]
	cellulose	30% AcOH			
Luteolin 7-rhamnoside	polyamide	CHCl_3_:MeOH:MEK:AcAc (9:4:2:1 *v/v/v/v*); To:MeOH:MEK:BuOH (300:150:150:3 *v/v/v/v*)			
Luteolin 3’-glucoside	silica gel 60 F_254_	MeOH:H_2_O (15:5 *v/v*); CHCl_3_:MeOH (15:5 *v/v*); 15% AcOH	NP/PEG; UV, 366 nm	*Phlomis persica*	[27]
Luteolin 6,8-dihexoside					
Luteolin 7-rutinoside				*Ph. elliptica*	
Luteolin 7-glucoside	silica gel	EtOAc:FA:H_2_O (18:1:1 *v/v/v*)	NP/PEG; UV, 366 nm	*Carduus acanthoides*	[28]
	polyamide plates	EtOAc:FA:AcOH:H_2_O (100:10:10:13 *v/v/v/v*)			
Luteolin	silica gel 60 F_254_	EtOAc:Ace:FA (8:1:1 *v/v/v*); EtOAc:H_2_O:AcOH:FA (10:3:1:1 *v/v/v/v*)	NP; UV, IR, NMR	*Leontopodium alpium*	[29]
Luteolin 7,4’-diglucoside					
Luteolin 6-hydroxy-7-glucoside					
Luteolin 4’-glucoside					
Luteolin 3’-glucoside					
Luteolin 7-glucoside					
Luteolin	silica gel 60 F_254_	To:Et_2_O:AcOH (60:40:10 v/v/v); EtOAc:AcOH:FA:H_2_O (100:11:11:26 v/v/v/v)	NP/PEG; UV, 366 nm	*Matricaria recutita, Achillea millefolium, Thymus vulgaris, Salvia officinalis*	[30]
Luteolin 7-glucoside					

Abbreviations: AcAc, acetylacetone; Ace, acetone; AcOH, acetic acid; BuOH, butanol; CHCl_3_, chloroform; DI, 1,4-dioxane; Et_2_O, diethyl ether; EtOAc, ethyl acetate; FA, formic acid; FBS, Fast Blue B Salt; Hx, hexane; MeOH, methanol; MEK, methyl ethyl ketone; NP, 1% methanolic diphenylboric acid-β-ethylamino ester - natural product reagents; PEG, 5% ethanolic polyethylene glycol 4000; To, toluene; UV, ultraviolet spectroscopy; MS, mass spectrometry; NMR, nuclear magnetic resonance; IR, infrared.

**Table 3:** High performance thin layer chromatography in the analysis of luteolin derivatives.

Luteolin Derivative	Stationary Phase	Mobile Phase	Detection	Analyzed Species	Ref.
Luteolin	HPTLC silica gel 60 F_254_	Hx:EtOAc:AcOH (31:14:5 *v/v/v*); To:DI:AcOH (90:25:4 *v/v/v)*	FBS; UV, 254, 366 nm	*Artemisia annua*	[25]
	HPTLC diol F_254_S	CHCl_3_:Hx:EtOAc (34:4 *v/v/v*)	2% AlCl_3_; UV, 366 nm	*Oxytropis glabra*	[31]
	HPTLC silica gel 60 RP-18W	BuOH:MeOH:H_2_O (300:50:50 *v/v/v*); 15% AcOH	NPR/PEG; UV, 366 nm		
	HPTLC silica gel 60 F_254_	EtOAc:MeOH:FA:H_2_O (20:3:1:2 *v/v/v/v*)	MeOH:H_2_SO_4_ (95:5 *v/v*); UV, 254, 366 nm	*Asparagus racemosus,* *Withania somnifera,* *Vitex negundo,* *Plumbago zylenica,* *Butea monosperma,* *Tephrosia purpurea*	[32]
	HPTLC silica gel 60 F_254_	To:EtOAc:FA (10:9:1 *v/v/v*)	UV, 254 nm	*Cardiospermum halicacabum*	[33]
	HPTLC silica gel 60 F_254_	DCM:MeOH (70:30 *v/v*)	UV, 254 nm; NMR	*Satureja montana*	[34]
	HPTLC silica gel G_60_ F_254_	To:EtOAc:FA (6:4:1 *v/v/v*)	UV, 349 nm	*Hygrophila spinosa*	[35]
	HPTLC silica gel 60 F_254_	EtOAc:MeOH:H_2_O:AcOH (3:1:1:1 *v/v/v/v*)	UV, 254, 366 nm	*Foeniculum vulgare*, *Cuminum cyminum,* *Apium graveolens,* *Petroselinum crispum,* *Anethum graveolens,* *Ammi majus*	[36]
	HPTLC silica gel 60 F_254_	To:EtOAc:FA (3:3:0.8 *v/v/v*)	MeOH:H_2_SO_4_ (90:10 *v/v*); UV, 254 nm	*Saraca asoca*	[37]
	HPTLC silica gel 60 F_254_	To:EtOAc:FA (6:4:0.3 *v/v/v*)	NP/PEG; UV, 366 nm	*Premna mucronata*	[38]
	HPTLC silica gel G_60_ F_254_	To:EtOAc:FA (10:9:1 *v/v/v*)	UV, 254, 366 nm	*Anisochilus carnosus*	[39]
	HPTLC silica gel 60	*n*Hx:EtOAc:FA (30:20:1.5 *v/v/v*)	NP/PEG; UV, 349 nm	*Satureja hortensis*	[40]
	HPTLC silica gel 60 F_254_	*n*Hx:EtOAc:AcOH (5:3:1 *v/v/v*)	UV	Propolis	[41]
	HPTLC silica gel 60 GF_254_	To:EtOAc:FA:MeOH (3:3:0.8:0.2 *v/v/v/v*)	UV, 254, 365nm	*Eclipta alba*	[42]
	HPTLC silica gel 60 F_254_	To:EtOAc:FA (5:3:1 *v/v/v*)	NP; UV, 366, 254 nm	*Vitis vinifera*	[43]
Luteolin 6-glucoside Luteolin 8-glucoside	HPTLC silica gel 60 F_254_	EtOAc:FA:AcOH:H_2_O (100:11:11:27 *v/v/v/v*)	NP/PEG; UV, 366 nm	*Clinacanthus nutans*	[44]
	HPTLC silica gel 60 F_254_	EtOAc:FA:H_2_O (82:9:9 *v/v/v*)	NP/PEG; UV, 366 nm	*Passiflora alata,* *P. edulis*	[45]
	Nano-DUASIL silica gel 60	THF:To:FA:H_2_O (16:8:2:1 *v/v/v/v*)	UV, 350 nm	*Phyllostachys pubescens*	[46]
Luteolin glucoside	HPTLC silica gel 60 F_254_	EtOAc:FA:AcOH:H_2_O (100:11:11:26 *v/v/v/v*)	NP/PEG; UV, 366 nm	*Equisetum arvense*	[47]
	HPTLC silica gel 60 F_254_	EtOAc:AcOH:FA:H_2_O (10: 1.1:1.1:2.6 *v/v/v/v*)	UV, 254, 366 nm	*Aerva javanica*	[48]
Luteolin	NP-HPTLC silica gel	EtOAc:FA:AcOH:H_2_O (100:11:11:27 *v/v/v/v*)	H_2_O solution of 4% Al_2_(SO_4_)_3_; UV, 365 nm	*Apis mellifera*, honey	[49]
		To:EtOAc:AcOH (50:45:5 *v/v/v*)			
	HPTLC silica gel 60 F_254_	To:EtFo:FA (6:4:1 *v/v/v*)	NP; UV, 254, 366 nm	*Potentilla grandiflora,* *P. recta,* *P. anserina,* *P. fruticose,* *P. rupestris,* *P. thuringiaca*	[50]
Luteolin 7-glucoside	modified HPTLC silica gel 60 F_254_ with CN, NH_2_	To:EtFo:FA (7:5:1 *v/v/v*)			
	HPTLC diol F_254_	EtOAc:MEK:DIPE:FA (3:10:4:1 *v/v/v/v*)			
Luteolin 7-glucoside	HPTLC silica gel 60	EtOAC:DCM:AcOH:FA:H_2_O (100:25:10:10:11 *v/v*)	NP; UV, 366 nm	*Lavandula stoechas*	[51]
	HPTLC silica gel 60 F_254_	EtOAc:AcOH:FA:H_2_O (100:11:11:26 *v/v/v/v*)	NP/PEG; UV, 254, 366 nm	*Stachys sylvatica,* *S. recta*	[52]
Luteolin 7-rutinoside	HPTLC NH_2_	Ace:AcOH (85:15 *v/v*)	MeOH:AlCl_3_ (98:2 *v/v*), *bis*-diazodized sulfanilamide; UV-VIS, 365 nm IR, MS, NMR	*Mentha piperita*	[53]
	HPTLC RP-18W	H_2_O:MeOH (60:40 *v/v*)			
Luteolin 7-glucuronide	HPTLC silica gel 60	Et:Ace:H2O:FA (55:25:10:10 *v/v/v/v*)	NP/PEG; UV, 365 nm	*Mentha piperita,* *Melissa officinalis,* *Salvia officinalis*	[54]
Luteolin 7-glucoside	HPTLC NH_2_	Ace:AcOH (85:15 *v/v*)			
Luteolin 7-rutinoside	HPTLC NH_2_	Ace:AcOH (85:15 *v/v*)	MeOH:AlCl_3_ (98:2 *v/v*), NP/PEG; UV, 365 nm	*Thymus vulgaris,* *Th. serpyllum,* *Majorana hortensis,* *Mentha piperita*	[55]
Luteolin 7-glucoside					
Luteolin 7-glucuronide		Ace:FA (85:15 *v/v*)			[56]
	HPTLC silica gel 60	DIPE:Ace:H_2_O:FA (50:30:10:10 *v/v/v/v*)			
Luteolin	HPTLC silica gel 60 F_254_	pre-develop: CHCl_3_:MeOH (1:1 *v/v*); *n*Hx:EtOAc:FA (20:19:1 *v/v/v*)	NP: white light, 254, 366, 330 nm; PEG: 254, 297, 340, 366, 430 nm; paraffin–*n*Hx: 254, 320, 360, 366, 400 nm; ESI-MS/MS	*Rosmarinus officinalis*	[57]
Luteolin acetyl hexuronide				*Salvia officinalis*	
Luteolin 7-rutinoside					
Luteolin acetyl hexoside					
Luteolin hexuronide					
Luteolin 3’,7-diglucoside	HPTLC silica gel 60 F_254_	EtOAc:MeOH:AcOH:FA:H_2_O (30:1:2:1:3 *v/v/v/v/v*)	NP; UV, 366 nm	*Colocasia esculenta*	[58]
Luteolin 4’-glucoside					
Luteolin 7-glucoside					
Luteolin 8-glucoside					
Luteolin 6-glucoside					
Luteolin 8-glucoside	HPTLC RP C_18_	FA:MeOH:H_2_O (0.5:6.65:2.85 *v/v/v*)	ESI-MS/MS, 254, 366 nm	*Cyclanthera pedata*	[59]
	HPTLC silica gel F_254_	To:EtOAc:FA (6:5:1 *v/v/v*)			

Abbreviations: Ace, acetone; AcOH, acetic acid; AlCl_3,_ aluminium trichloride; Al_2_(SO_4_)_3_, aluminium sulfate; BuOH, butanol; CHCl_3_, chloroform; DCM, dichloromethane; DI, 1,4-dioxane; DIPE, diisopropyl ether; ESI-MS/MS, electrospray ionization combined with tandem mass spectrometry; EtFo, ethyl formate; EtOAc, ethyl acetate; Et, ether; FA, formic acid; H_2_SO_4_, sulfuric acid; Hx, hexane; MeOH, methanol; MEK, methyl ethyl ketone; *n*Hx, *n*-hexane; NP, 1% methanolic diphenylboric acid-β-ethylamino ester - natural product reagents; neurotransmitters; PEG, 5% ethanolic polyethylene glycol 4000; THF, tetrahydrofuran; To, toluene; UV-VIS, ultraviolet-visible spectroscopy; ESI-MS, electrospray ionization combined with mass spectrometry.

**Table 4:** High performance liquid chromatography/ultra performance liquid chromatography in the analysis of luteolin derivatives.

Luteolin Derivative	Stationary Phase/Column	Mobile Phase	Conditions	Detection	Analyzed Species	Ref.
Luteolin	ODS	0.2% PA:H_2_O (A), MeOH (B)	injection volume: 20 μL; flow rate: 1.0 mL/min	DAD, 360 nm	Honey	[97]
	ODS C_18_	0.2% PA:H_2_O (58:41 *v/v*)	injection volume: 50 µL; flow rate: 1.0 mL/min	UV, 350 nm	*Chrysanthemum morifolium*	[98]
	Kinetex C_18_	ACN (A), 0.05% TFA:H_2_O (B)	flow rate: 0.8 mL/min	UV, 254 nm	*Enhalus acoroides*	[99]
	ODS Hypersil C_18_	MeOH:ACN (1.25:1 *v/v*) (A), 0.5% AcOH:H_2_O (B)	flow rate: 0.8 mL/min	MS	*Perilla frutescens*	[100]
	Zorbax SB-C_18_	PA:H_2_O pH 4.0 (A), ACN (B)	injection volume: 50 µL; flow rate: 0.6 ml/min; 25 °C	DAD, 330 nm	*Vernonia condensata*	[101]
	ODS C_18_	MeOH (A), 0.05% TFA:H_2_O (B)	injection volume: 20 μL; flow rate: 1.0 mL/min	UV, 280 nm	*Corchorus olitorius*	[102]
	RP	0.5% PA:H_2_O (A), ACN (B)	injection volume: 20 μL; flow rate: 0.8 mL/min; at 35 ℃	Triple-TOF-MS	*Veronicastrum latifolium*	[103]
	RP C_18_	1% FA:H_2_O (A), 40% solvent A:ACN (B)	flow rate: 0.5 mL/min; 25 °C	DAD-ESI-MS/MS	*Olea europaea,* olive oil	[104]
	Discovery HS C_18_	2% AcOH (A), ACN (B)	injection volume: 20 μL; flow rate: 0.8 mL/min	DAD 280, 320 nm	*Agastache foeniculum*	[105]
	Ultrasphere 5 C_18_	H_2_O (A), ACN (B)	injection volume: 10 μL; flow rate: 1 mL/min; 25 °C	ESI-MS; 350 nm	*Rosa rugosa*	[106]
Luteolin 6-glucoside	Vydac RP C_18_	0.05% TFA:H_2_O (A), 0.038% TFA:ACN (*v/v*) (B)	injection volume: 10 μL flow rate: 1 mL/min; 36 °C	UV, 342 nm	*Ficaria verna*	[107]
Luteolin 8-glucoside	RP C_18_	0.5% FA:H_2_O (A), ACN (B)	injection volume: 20 µL; flow rate: 0.5 mL/min; 23 °C	DAD, 254, 340 nm	*Jatropha gossypiifolia, J. mollissima*	[108]
Luteolin 7-glucoside	Hypersil gold C_18_	0.1% FA:H_2_O (A), 0.1% FA:MeOH (B)	injection volume: 10 µL; flow rate: 0.35 mL/min; 38 °C	MS	*Rhoeo discolor*	[109]
	Thermo C_18_	0.3% FA:H_2_O (A), ACN (B)	injection volume:10 μL; flow rate: 1.0 mL/min; 30 °C	DAD-Q- Orbitrap-MS	*Epimedium brevicornum,* *Anacyclus pyrethrum,* *Lycium barbarum,* *Cuscuta australis*	[110]
Luteolin	Eclipse XDB C_18_	0.025% AcOH:H_2_O (A), 5% Ace:ACN (B)	30 °C	ESI-MS/MS	*Olea europaea,* olive oil	[111]
Luteolin 7-glucoside	Eclipse XDB C_18_	1% PA:H_2_O (A), ACN (B)	injection volume: 10 μL; flow rate: 1 mL/min; 25 °C	DAD-ESI-QTOF-MS/MS	*Verbascum ovalifolium*	[112]
Luteolin 7-rutinoside	Eclipse XDB C_18_	ACN (A), 0.2% FA (B)	flow rate: 0.3 mL/min; 30 °C	DAD, 230, 254, 290, 334 nm; MS/MS; NMR	*Ligustrum vulgare*	[26]
Luteolin 7-rhamnoside						
Luteolin	Gemini C_18_	0.5% AcOH:H_2_O (A), ACN (B)	25 °C	ESI-QTOF-MS	*Olea europaea*	[78]
Luteolin 7,4-diglucoside						
Luteolin 7-rutinoside						
Luteolin 4-glucoside						
Luteolin 3-glucoside						
Luteolin 7-glucoside						
Luteolin 6-glucoside	Spherisorb S5 ODS-2	5% FA:H_2_O (A), MeOH (B)	flow rate: 1 mL/min	DAD, 280 nm	*Cymbopogon citratus*	[79]
Luteolin 7*-*rhamnoside						
Luteolin 7-glucoside						
Luteolin 6-glucosyl-8-arabinoside						
Luteolin 2’’-feruloylhexosyl-6-hexoside	Spherisorb S3 ODS-2 C_18_	0.1% FA:H_2_O (A), ACN (B)	flow rate: 0.5 mL/min; 35 °C	DAD-MS; 280, 370 nm	*Arenaria montana*	[66,67]
Luteolin 6-glucoside						
Luteolin	Kinetex 100 C_18_	1% FA:H_2_O (A), 1% FA:ACN (B)	flow rate: 1 mL/min; 30 °C	ESI-QTOF-MS;	*Rosmarinus officinalis*	[82]
Luteolin 7-rutinoside						
Luteolin 3-glucuronide						
Luteolin 3’-(2’’-acetyl)-glucuronide	Superschera 100 RP C_18_			DAD, 324 nm		
Luteolin 6-hydroxy-7-glucoside						
Luteolin hexosyl-hexoside-malylester	AquasilW C_18_	TFA solution (pH 2.8) (A), ACN (B)	flow rate: 1.0 mL/min; 15 °C	UV/DAD, 340 nm	*Dianthus versicolor*	[88]
Luteolin 6-glucosyl-7-galactoside						
Luteolin 6-glucosyl-7-rutinoside						
Luteolin 6-glucosyl-7-rhamnosyl-galactoside						
Luteolin 7-apiofuranosyl (1→2)-glucopyranoside	Eclipse Plus C_18_	0.1% FA:MeOH (A), 0.1% FA:H_2_O (B)	injection volume: 10 μL; isocratic mixture flow rate: 0.3 mL/min	QTOF-MS/MS	*Apium graveolens*	[80]
Luteolin 7-glucopyranoside						
Luteolin 7-[apiofuranosyl (1→2)-(6’’-malony)]-glucopyranoside						
Luteolin 7-(6-rhamnosyl)hexoside	Kinetex C_18_	1% FA (A), ACN (B)	injection volume: 8 μL; flow rate: 0.8 mL/min	DAD-ESI-MS; 340 nm	*Phoenix dactylifera*	[94]
Luteolin 7-(2-rhamnosyl)hexoside						
Luteolin 7-(2-hexosyl[6-sulfate])hexoside						
Luteolin 7-hexosyl(6-sulfate)						
Luteolin	Gemini C_18_	0.1% FA:H_2_O (A), 0.1% FA:ACN (B)	0-60 min, 10–60% B; flow rate: 1 mL/min	DAD-ESI-MS/MS; 210, 270, 310 and 350 nm	*Achillea moschata*	[96]
Luteolin 7-glucoside						
Luteolin 7-glucoside	Synergy Polar RP 80Å, LiChroCART 4-4 with guard column LiChrospher 100 C_18_	0.9% FA:0.1% AcOH:1.5% BuOH:H_2_O (A), 30% MeOH:0.9% FA:0.1% AcOH:ACN (B), MeOH (C)	injection volume: 5 μL; flow rate: 1.0 mL/min; 45 °C	PAD; MS; UV; IR; NMR	*Leontopodium alpium*	[29]
Luteolin 7,4’-diglucoside						
Luteolin 6-hydroxy-7-glucoside						
Luteolin 4’-glucoside						
Luteolin 3’-glucoside						
Luteolin 7-diglucuronide	Eclipse Plus C_18_	1% FA:H_2_O:ACN (A), ACN (B)	injection volume: 20 μL; flow rate: 0.5 mL/min	DAD; UV-VIS, 190-450 nm; ESI-TOF-MS; ESI-IT-MS/MS	*Lippia citrodora*	[113]
Luteolin 5-rutinoside	Luna C_18_	0.1% FA:H_2_O (A), ACN (B)	injection volume: 5 μL; flow rate: 1.0 mL/min	DAD, 200-400 nm; ESI-MS	*Nerium indicum*	[95]
Luteolin 7-rutinoside						
Luteolin diglucoside	Luna C_18_	1% FA:H_2_O (A), ACN (B)	injection volume: 20 μL; flow rate: 1 mL/min	PDA-ESI-MS/MS	*Taraxacum officinale*	[114]
Luteolin 7-rutinoside						
Luteolin 7*-*glucoside						
Luteolin	Gemini RP C_18_	2% AcOH:H_2_O (A), ACN (B)	---	ESI-MS; 257, 278 and 340 nm	*Pistacia atlantica*	[85]
Luteolin 2’’-Galloyl-4’-glucoside						
Luteolin 4’-glucoside						
Luteolin 6-methoxy-8-arabinosyl-7-glucoside	Symmetry Shield Waters RP_18_	0.2% FA:H_2_O (A), ACN (B)	flow rate: 1.2 mL/min	UV/PAD-MS; UV; 200–600 nm	*Saccharum officinarum*	[115]
Luteolin 8-glucoside	Symmetry Shield RP_18_			UV/PAD		
Luteolin 8-rhamnosyl-7-rhamnoside						
Luteolin 8-glucoside	Kinetex PFP	0.8% AcOH:H_2_O (A), ACN (B)	injection volume: 10 μL; flow rate: 0.7 mL/min; 40 °C	UV/DAD, 330 nm	*Clinacanthus nutans*	[44]
Luteolin	Eclipse XDB C_18_	MeOH (A), 0.2% FA:H_2_O (B)	flow rate: 0.8 mL/min; 30 °C	DAD-MS/MS; UV; 254, 360 nm	*Securigera securidaca*	[75]
Luteolin 7-glucoside						
Luteolin 7-glucuronyl-3-glucoside						
Luteolin 6-glucoside						
Luteolin 6-glucosyl-2’’-rhamnoside						
Luteolin 6-glucosyl-4’-glucoside						
Luteolin 3’-glucoside						
Luteolin hexosyl-rhamnoside	Spherisorb S3 ODS-2 C_8_	0.1% FA:H_2_O (A), ACN (B)	flow rate: 0.5 mL/min; 35 °C	DAD-MS; 280, 370 nm	*Coriandrum sativum*	[77]
Luteolin 7-rutinoside						
Luteolin 7-glucoside						
Luteolin	XDB C_18_	1% FA:H_2_O (A), ACN (B)	injection volume: 100 µL; flow rate: 4 mL/min	NMR; MS	*Casimiroa edulis*	[116,117]
Luteolin 6-glucosyl-8-arabinoside						
Luteolin 7-glucoside						
Luteolin 6-arabinosyl-8-glucoside						
Luteolin 3-glucopyranoside	PLRP-S 100Å	0.1% FA:H_2_O (A), ACN (B)	injection volume: 100 µL; flow rate: 1.5 mL/min	MS	*Thymus alternans*	[93]
Luteolin 7-glucopyranoside						
Luteolin 7-rutinoside						
Luteolin methoxy-hexoside						
Luteolin 4’-glucopyranoside	XDB C_18_	1% FA:H_2_O (A), ACN (B),	injection volume: 100 µL; flow rate: 4 mL/min			
Luteolin 6-hydroxy-7-glucoside	Gemini C_18_	0.1% FA:H_2_O (A), ACN (B)	flow rate: 0.8 mL/min	UV-VIS, 200-400 nm; MS; DAD; NMR	*Athrixia phylicoides*	[118]
Luteolin	Syncronis C_18_	0.1% AcOH:H_2_O (A), ACN (B)	injection volume: 5 μL; flow rate: 0.3 mL/min	DAD-MS/MS	Honey	[86]
Luteolin 7-rhamnoside						
Luteolin	HSS T3	0.1% FA:H_2_O (A), 0.1% FA:ACN (B)	injection volume: 3.1 µL; flow rate: 0.15 mL/min	PDA-MS	*Phoenix dactylifera*	[74]
Luteolin rhamnosyl hexoside						
Luteolin rhamnosyl dihexoside						
Luteolin diglucuronide	S3 ODS-2 C_18_	0.1% FA:H_2_O (A), ACN (B)	flow rate: 0.5 mL/min; 35 °C.	DAD-ESI-MS; 370, 330 and 280 nm	*Thymus pallescens, Saccocalyx satureioides,*	[119,120]
Luteolin 7-glucuronide						
Luteolin 7-rutinoside						
Luteolin 7-glucoside					*Ptychotis verticillata*	
Luteolin 6-glucoside					*Coleostephus myconis*	
Luteolin	Kinetex C_18_	ACN (A), 0.1% FA:H_2_O (B)	injection volume: 10 μL; flow rate: 0.4 mL/min	ESI-MS	*Lathyrus pratensis*	[121]
Luteolin rutinoside						
Luteolin hexoside						
Luteolin 6-hexoside					*L. aureus*	
Luteolin 7-glucoside	Luna C_18_	5% FA:H_2_O (A), MeOH (B)	injection volume: 100 µL; flow rate: 1 mL/min; 35 °C	DAD, 200, 600 nm	*Thymus pulegioides*	[81]
Luteolin hexuronide		0.1% FA:H_2_O (A), MeOH (B)	injection volume: 10 µL; flow rate: 0.5 mL/min; 40 °C	ESI-MS/MS		
Luteolin	Eclipse Plus C_18_	0.5% AcOH:H_2_O (A), ACN (B)	injection volume: 5 µL; flow rate: 0.5 mL/min	DAD-QTOF-MS; 325-371 nm	*Ficus carica*	[87]
Luteolin hexosyl-pentoside						
Luteolin 6-glucoside						
Luteolin 8-glucoside						
Luteolin 7-glucoside						
Luteolin (3-hydroxy-3-methylglutaroyl)-X’’-deoxyhexosyl-hexoside	ODS-2 C_18_ with ODS-2 C_18_ guard cartridge	1% FA:H_2_O (A), MeOH (B)	flow rate: 0.2 mL/min; 25 °C	PDA-ESI-MS	*Urtica membranacea*	[122]
Luteolin 6-hexoside						
Luteolin 6-rutinoside						
Luteolin dihexoside						
Luteolin	Syncronis C_18_	0.01% AcOH:H_2_O (A), ACN (B)	inaction volume: 5 μL; flow rate: 0.25 mL/min	MS	*Capsicum annuum*	[76]
Luteolin 6,8-dihexoside						
Luteolin 6-hexosyl-8-pentoside						
Luteolin 6-pentosyl-8-hexoside						
Luteolin 6-hexoside						
Luteolin 8-hexoside						
Luteolin 7-(2’’-pentosyl-4’’-hexosyl)hexoside						
Luteolin 7-(2’’-pentosyl)hexoside						
Luteolin 7-glucoside						
Luteolin 7-[2’’-(5’’’-sinapoyl)pentosyl]hexoside						
Luteolin 7-(2’’-pentosyl-4’’-hexosyl- 6’’-malonyl)hexoside						
Luteolin 7-(2’’-pentosyl-6’’-malonyl) hexoside						
Luteolin 7-[2’’-(5’’’-sinapoyl)pentosyl- 6’’-malonyl]hexoside						
Luteolin	Purospher star C_18_	10% FA:H_2_O (A), ACN (B)	25 °C	DAD-ESI-MS; 320-280 nm	*Citrus aurantifolia*	[123]
Luteolin 6,8-diglucoside						
Luteolin 8-glucoside						
Luteolin 7-rutinoside						
Luteolin	Zorbax SB-C_18_	AFNH_4_:H_2_O:ACN:FA (A), AFNH_4_:H_2_O:ACN:FA (B)	injection volume: 5µL; flow rate: 1.0 mL/min	ESI-MS; 325 nm	*Caucalis platycarpos*	[124]
Luteolin 7*-*glucoside						
Luteolin 7-rutinoside						
Luteolin 7-glucoside	Beta-Basic C_18_	5% FA:ACN (A); 5% FA:H_2_O (B)	injection volume: 20 µL; flow rate: 0.9 mL/min	UV, 280 nm	*Melissa officinalis*	[54]
Luteolin 7-glucuronide					*Mentha piperita,* *Salvia officinalis*	
Luteolin 7-rutinoside						
Luteolin Luteolin 7-glucuronide Luteolin 7-rhamnosyl-hexoside Luteolin 7-rutinoside Luteolin 7-glucoside Luteolin 7-glucuronide Luteolin 7-rutinoside Luteolin diglucoside	UPLC BEH C_18_ and a Acquity UPLC BEH C_18_ VanGuardTM pre-column	0.1% AcOH:H_2_O (A), 0.1% AcOH:MeOH (B)	injection volume: 5 µL; flow rate: 0.5 mL/min	DAD-ESI-QTOF-MS; 370 nm	*Lactuca sativa*	[72]
	HSS T3	0.1% FA:H_2_O (A), 0.1% FA:ACN (B)	injection volume: 3 µL; flow rate: 0.4 mL/min	IMS-QTOF-MS		[73]
Luteolin hexoside	Hypersil Gold C_18_	0.1% FA:H_2_O (A), ACN (B)	---	DAD-ESI-MS	*Salvia elegans,* *S. greggii,* *S. officinalis*	[125,126]
Luteolin hydroxy-glucuronide						
Luteolin 7-glucoside						
Luteolin 7-glucuronide						
Luteolin malonyl-hexoside						
Luteolin glucuronide						
Luteolin glucoside			flow rate: 0.2 mL/min		*Thymus barona*, *T. pseudolanuginosus*, *T. caespititius*	[126]
Luteolin rutinoside						
Luteolin 7-rhamnosyl(1-6)galactoside	Eclipse XDB-C_18_	0.1% FA:H_2_O (A), 0.1% FA:ACN (B)	flow rate: 0.5 mL/min; 25 °C	MS	*Filago germanica*	[91]
Luteolin 4’-glucuronide	C_18_μ-Bondapak RP_18_	MeOH:H_2_O (42:58 *v/v*)	flow rate: 2.0 mL/min	NMR	*Galactites elegans*	[90]
Luteolin	Kinetex 100 A C_18_	1% FA:H_2_O (A), ACN (B)	injection volume: 5 µL; flow rate: 0.8 mL/min; 25 °C	DAD, 340 nm	*Allophylus africanus*	[83,127]
Luteolin 3’-7-diglucoside						
Luteolin 7-glucoside						
Luteolin 6-(2-rhamnosyl)-hexoside				DAD-ESI-MS/MS; 280, 340 nm		
Luteolin (pentosyl)-hexoside						
Luteolin hexoside						
Luteolin 6-glucoside	ODS2 C_8_	0.1% FA:H_2_O (A), ACN (B)	flow rate: 0.5 mL/min; 35 °C	DAD-ESI-MS; 280, 370 nm	*Achillea millefolium*	[68,69]
Luteolin 6-hexosyl-8-pentoside	S3 ODS-2 C_18_	0.1% FA:H_2_O (A), ACN (B)	flow rate: 0.5 mL/min; 35 °C	DAD-MS; 280, 370 nm	*Cymbopogon citratus*	[70,71]
Luteolin 2’’-deoxyhexosyl-6-glucoside						
Luteolin 6-glucoside						
Luteolin 6-pentosyl-8-pentoside						
Luteolin-7-rhamnoside						
Luteolin 7-glucoside						
Luteolin 2’’-deoxyhexosyl-pentoside						
Luteolin 6-pentoside						
Luteolin 2’’-deoxyosyl-6-(6-deoxy- pento-hexosuloside						
Luteolin	C_18_	0.005 % FA:H_2_O (A), MeOH (B)	injection volume: 10 µL; flow rate: 0.5 mL/min; 30 °C	MS-Orbitrap	*Chrysanthemum trifurcatum*	[92]
Luteolin 8-glucoside						
Luteolin 4’-glucoside						
Luteolin 7-glucoside						
Luteolin 6-glucoside						
Luteolin 6-glucoside	HSS T3	0.1% FA:H_2_O (A), 0.1% FA:ACN (B)	injection volume: 3.1 μL; flow rate: 0.15 mL/min	PDA-MS	*Passiflora edulis*	[128]
Luteolin 8*-*glucoside						
Luteolin 6-deoxyhexosyl-8-pentoside						
Luteolin 6-fucoside						
Luteolin 8-deoxyhexoside						
Luteolin 6,8-diglucoside						
Luteolin 6,8-diglucoside	Kinetex C_18_	0.05% FA:H_2_O (A), 0.05% FA:ACN (B)	flow rate: 0.4 mL/min; 40 °C	MS/MS	*Eragrostis tef*	[84]
Luteolin 8-glucosyl-7-glucoside isomer						
Luteolin 6-glucosyl-7-glucoside isomer						
Luteolin 8-glucosyl-7-rhamnoside						
Luteolin 8-glucoside						
Luteolin 7-glucoside						
Luteolin 7-rhamnoside						
Luteolin 7-(6’’-syringly)glucosyl-6-glucoside						
Luteolin 7-(2’’-syringyl)arabinosyl-6*-*glucoside						
Luteolin 8-(6’’-diacetyl)glucoside						
Luteolin	Nucleosil 100-3.5 C_18_	FA:H_2_O (A), FA:ACN (B)	injection volume: 20 µL	MS	*Arum hygrophilum*	[129]
Luteolin 6-glucoside						
Luteolin 6-[6’’-glocosy-caffeoyl-glucopyranosyl(’’→2)-glucopyranoside	Eclipse Plus C_18_	0.2% FA:H_2_O (A), 0.2% FA:ACN (B)	30 °C	PDA-ESI-MS/MS	*Triticum aestivum*	[130]
Luteolin 6-glucopyranoside				UV, 350 nm		
Luteolin 7-glucoside	Syncronis C_18_	0.1% FA:H_2_O (A), ACN (B)	injection volume: 5 μL; flow rate: 0.25 mL/min	MS/MS	*Iris pumila,* *I. variegata,* *I. humilis*	[131]
Luteolin 6-glucoside						
Luteolin 7-(2’’-*p*-coumaroyl)-rhamnoside						
Luteolin	Luna Omega Polar C_18_ with Polar C_18_ Security Guard cartridge	0.1% FA:H_2_O (A), ACN (B)	flow rate: 0.4 mL/min	ESI-MS/MS; 320, 350 nm	*Parentucellia latifolia*	[132,133]
Luteolin hexoside						
Luteolin	Intersil ODS	ACN:H_2_O:FA (10:89:1 *v/v/v*) (A), ACN:H_2_O:FA (89:10:1 *v/v/v*) (B)	injection volume: 10 µL; flow rate: 0.5 mL/min; 40 ℃	DAD-MS/MS; 360 nm	*Verbascum eskisehirensis*	[134]
Luteolin glucoside						
Luteolin glucuronide						
Luteolin pentosyl-glucoside						
Luteolin 4’-glucoside	Kinetex RP C_18_	PA:H_2_O pH 3 (A), PA:ACN pH 3 (B)	injection volume: 1 µL; flow rate: 1.2 mL/min; 35 ℃	UV 300 nm	*Matricaria recutita*	[135]
Luteolin 7-*O*-glucuronide	C_18_	0.02% TFA:H_2_O (A), MeOH:ACN (3:7 *v/v*) (B)	injection volume: 2.5 µL; flow rate: 0.9 mL/min; 45 °C	DAD-ESI-MS 240, 254, 325 nm	*Lippia alba*	[136]
Luteolin glucoside	YMC-Triart C_18_	0.1% FA:H_2_O (A), 0.1% FA:ACN B)	injection volume: 10 µL; flow rate: 0.8 mL/min	DAD-ESI-MA 265, 280, 330, and 360 nm	*Chrysanthemum morifolium*	[137]
Luteolin 7-*O*-β-glucoside						
Luteolin glucuronide						
Luteolin malonylglucoside						
Luteolin						
Luteolin 7-diglucuronide	Eclipse XDB C_18_	0.03% PA:H_2_O (A), solvent A:ACN (1:9 *v/v*) (B)	injection volume: 20 μL; flow rate: 0.8 mL/min; 25 °C	DAD, 210, 250, 320, 350 and 370 nm	*Thymus pannonicus*	[138]
Luteolin 7-*O*-glucuronide						

Abbreviations: Ace, acetone; AcOH, acetic acid; ACN, acetonitrile; AFNH_4_, ammonium formate; BuOH, butanol; DAD, diode array detector; DAD-ESI-MS, diode array detector combined with electrospray ionization and mass spectrometry; DAD-ESI-MS/MS, diode array detector combined with electrospray ionization and tandem mass spectrometry; DAD-ESI-QTOF-MS, diode array detector combined with electrospray ionization and quadrupole – time of flight mass spectrometry; DAD-MS, diode array detector combined with mass spectrometry; DAD-MS/MS, diode array detector combined with tandem mass spectrometry; DAD-QTOF-MS, diode array detector combined with quadrupole – time of flight mass spectrometry; ESI-IT-MS/MS, electrospray ionization combined with ion trap and tandem mass spectrometry; ESI-MS, electrospray ionization combined with mass spectrometry; ESI-MS/MS, electrospray ionization combined with tandem mass spectrometry; ESI-QTOF-MS, electrospray ionization combined with quadrupole – time of flight mass spectrometry; FA, formic acid; IMS-QTOF-MS, ion-mobility spectrometry combined with quadrupole – time of flight mass spectrometry; MeOH, methanol; MS/MS, tandem mass spectrometry; PA, phosphoric acid; PAD, pulsed amperometric detection; PDA-ESI-MS/MS, pulsed amperometric detection combined with electrospray ionization and tandem mass spectrometry; PDA-ESI-MS, pulsed amperometric detection combined with electrospray ionization and mass spectrometry; PDA-MS, pulsed amperometric detection combined with mass spectrometry; TFA, trifluoroacetic acid; UV/PAD-MS, UV/pulsed amperometric detection combined with mass spectrometry.

**Table 5:** Liquid chromatography in the analysis of luteolin derivatives.

Luteolin Derivative	Stationary Phase	Mobile Phase	Conditions	Detection	Analyzed Species	Ref.
Luteolin	Zorbax SB C_18_ column with Security-Guard C_18_	MeOH (A), 0.5% AcOH:H_2_O (B)	flow rate: 1.0 mL/min; injection volume: 20 μL	MS/MS	*Abri herba,* *A. mollis*	[143]
	Inertsil ODS-3	0.1% FA:H_2_O (A), ACN (B)	flow rate: 0.5 mL/min; injection volume: 10 μL	MS/MS	*Castanea mollissima*	[144]
	RP C_18_	0.1% FA:H_2_O (A), 0.1% FA:ACN (B)	injection volume: 5 µL; flow rate: 0.3 mL/min; 40 °C	ESI-MS/MS	*Centaurea cyanus*	[145]
Luteolin 7-glucoside	Eclipse XDB C_18_	1% FA:H_2_O (A), MeOH (B)	injection volume: 5 mL; flow rate: 0.6 mL/min; 45 °C	MS/MS	*Plantago atrata,* *P. coronopus,* *P. holosteum,* *P. lanceolata,* *P. reniformis,* *P. schwarzenbergiana*	[146]
Luteolin	Zorbax Plus C_18_	0.1% FA:H_2_O (A), 0.1% FA:ACN (B)	injection volume: 1µL; flow rate: 0.4 mL/min	MS	*Matricaria recutita,* *Achillea millefolium,* *Thymus vulgaris,* *Salvia officinalis*	[30]
	Eclipse XDB C_18_	0.05% FA:H_2_O (A), MeOH (B)	flow rate: 1 mL/min	MS/MS	*Vitis vinifera*	[147]
Luteolin 7-apiosyl-glucoside	Symmetry C_18_	0.1% FA:H_2_O (A), 0.1% FA:ACN (B)	flow rate: 1.0 mL/min; 25 °C	DAD-ESI-MS; DAD, 350, 310, 270 nm; UV, 190-650 nm	*Apium graveolens*	[148]
Luteolin 7-glucoside						
Luteolin 7-malonyl-apiosyl-glucoside						
Luteolin 7-6’-malonyl-apiosyl-glucoside						
Luteolin 7-6’-malonyl-glucoside						
Luteolin 3’-glucoside	Zorbax SB C_18_	0.1% FA:H_2_O (A); 0.1% FA:ACN (B)	injection volume: 5 μL; flow rate: 0.3 mL/min; 25 °C	MS/MS; TQMS; ESI	*Phlomis persica*	[27]
Luteolin 6-glucoside						
Luteolin 6,8-dihexoside						
Luteolin 7-rutinoside					*Ph. eliptica*	
Luteolin 7-rutinoside	XTerra MS C_18_	ACN (A), 0.05% AcOH:H_2_O (B)	injection volume: 20 μL; flow rate: 1.0 mL/min	PDA-MS; NMR	*Sechium edule*	[149]
Luteolin 7-glucopyranoside						
Luteolin	Octadecyl silica gel ODS	H_2_O:MeOH:FA (89:10:1 *v/v/ v*) (A), MeOH:H_2_O:FA (89:10:1 *v/v/v*) (B)	flow rate: 1 mL/min; 40 °C	MS/MS	*Nepeta cilicica*	[150]
Luteolin glucuronide						
Luteolin glucoside						
Luteolin	Capcell Park C_18_	0.5% FA:H_2_O (A), 0.5% FA:ACN (B)	flow rate: 0.5 mL/min; 25 °C	MS/MS	*Humulus japonicus*	[151]
Luteolin dihexoside						
Luteolin 7-dihexoside						
Luteolin 7-rutinoside						
Luteolin glucoside						
Luteolin 7-acetylglucoside						
Luteolin 6-glucoside	Intersil ODS	ACN:H_2_O:FA (10:89:1 *v/v/v*) (A), ACN:H_2_O:FA (89:10:1 *v/v/v*) (B)	flow rate: 0.7 mL/min; 40 °C	MS/MS	*Achillea sivasica*	[152]
Luteolin glucoside						
Luteolin methoxy-2’’-pentosyl-6-hexoside	ODS2 C_8_	0.1% FA:H_2_O (A), ACN (B)	flow rate: 0.5 mL/min; 35 °C	DAD-ESI-MS; 280, 370 nm	*Geranium molle*	[68,70,153]
Luteolin 2’’-rhamnosyl-6’’-hexosyl-glucoside						
Luteolin 7-glucosyl-8-glucoside						
Luteolin 6-glucoside						
Luteolin 6-hexosyl-8-pentoside						
Luteolin acetylhexoside	Spherisorb S3 ODS2 C_18_	0.1% FA:H_2_O (A), ACN (B)	flow rate: 0.5 mL/min; 35 °C	DAD-ESI-MS/MS	*Achillea millefolium*	[120,154]
Luteolin 6-glucoside						
					*Coleostephus myconis*	[120]
Luteolin 3-glucuronide					*Rosmarinus officinalis*	[155]
Luteolin glucuronide						
Luteolin hexosyl-pentoside	Zorbax RP C_18_	0.1% FA:H_2_O (A), 0.1% FA:ACN (B)	injection volume: 5 μL; flow rate: 0.4 mL/min; 35 °C	QTOF	*Ficus carica*	[156]
Luteolin 7-glucoside						
Luteolin 7-hexoside						
Luteolin 6-hexoside						
Luteolin 8-glucoside						
Luteolin 6-hexosyl-8-acetyl-hexoside	ODS2	H_2_O:ACN:FA	injection volume: 20 μL; flow rate: 0.8 mL/min	MS/MS	*Oxalis pes-caprae*	[157]
Luteolin 8-glucosyl-7,3’-dimethoxyl- 2’’-*O*-glycoside						
Luteolin 8-glucosyl-7,3’-dimethoxyl- 6-desoxyhexoside						
Luteolin 6-glucoside	Hydro RP	H_2_O (A), MeOH (B), 5% AcOH:MeOH (C)	flow rate: 1 mL/min	MS/MS	*Passiflora morifolia*	[158]
Luteolin 8*-*glucoside						
Luteolin	Luna C_18_	0.1% FA:H_2_O (A), 0.1% FA:ACN (B)	flow rate: 0.4 mL/min	DAD, 280, 320, 365 nm; MS; MS/MS	*Theobroma cacao*	[141]
Luteolin 6-glucoside						
Luteolin 8*-*glucoside						
Luteolin 7-glucoside						
Luteolin 6-glucosie	XBridge C_18_	0.3% FA:H_2_O (A), MeOH (B)	injection volume: 1 μL; flow rate: 1 mL/min; 40 °C	MS/MS; ESI-MS	*Lophatherum gracile*	[140]
Luteolin 7-glucoside						
Luteolin	Symmetry C_18_	0.1% FA:H_2_O (A), 0.1% FA:ACN (B)	flow rate: 1.0 mL/min; 25 °C	DAD-ESI-MS; 350, 310, 270 and 520 nm	*Chrysanthemum morifolium*	[142]
Luteolin glucuronyl-hexoside						
Luteolin 7-pentosyl-hexoside						
Luteolin 7-rutinoside						
Luteolin 7-glucoside						
Luteolin 7-glucuronide						
Luteolin glucoside						
Luteolin 7-malonyl-6’’-glucoside						
Luteolin 7-acetyl-6’’-glucoside						
Luteolin 7-dihexoside						
Luteolin	Acquity BEH C_18_	0.1% FA:H_2_O (A), 0.1% FA:ACN (B)	flow rate: 0.3 mL/ min; 40 °C	QTOF-MS/MS	*Ageratum conyoides*	[159]
Luteolin7-glucuronide						
Luteolin hexoside	Zorbax SB C_18_	0.5% FA:H_2_O (A), ACN (B)	injection volume: 5 μL; flow rate: 0.4 mL/min	MS/MS	*Matricaria recutita*	[160]
Luteolin hexoside	Hypersil gold C_18_	1% FA:H_2_O (A), 0.1% FA:ACN (B)	flow rate: 1 mL/min	MS/MS	*Cecropia obtusa*	[161]
Luteolin hexosyl-deoxy-hexose						
Luteolin dihexoside	Spherisorb S3 ODS-2 C_18_	0.1% FA:H_2_O (A), ACN (B)	flow rate: 0.5 mL/min; 35 °C.	DAD-ESI-MS/MS	*Cotula cinerea*	[120,162]
Luteolin pentosyl-hexoside						
Luteolin 7-glucoside						
Luteolin malonyl-hexoside						
Luteolin	Zorbax SB C_18_	0.1% FA:AFNH_4_ (A), 0.1% FA:ACN (B)	injection volume: 1 mL; flow rate: 0.2 mL/min	ESI-MS/MS	*Ocimum sanctum*	[163]
Luteolin 8-glucoside						
Luteolin 5-glucopyranoside						

Abbreviations: AcOH, acetic acid; ACN, acetonitrile; AFNH_4_, ammonium formate; DAD-ESI-MS, diode array detector combined with electrospray ionization and mass spectrometry; DAD, diode array detector; DAD-ESI-MS/MS, diode array detector combined with electrospray ionization and tandem mass spectrometry; ESI, electrospray ionization; ESI-MS, electrospray ionization combined with mass spectrometry; FA, formic acid; MeOH, methanol; MS/MS, tandem mass spectrometry; PDA-MS, pulsed amperometric detection combined with mass spectrometry; QTOF, quadrupole time-of-flight; TQMS, triple quadrupole mass spectrometer.

**Table 6:** Gas chromatography in the analysis of luteolin and its derivatives.

Luteolin Derivative	Column	Derivatization	Conditions	Detection	Analyzed Species	Ref.
Luteolin	HP-5-MS	1% TMCS:BSTFA	carrier gas: He; injector temperature: 250 °C A: (column temperature) 5 min, 170°C; 3 °C/min, 170–255 °C; 1 min, 255 °C; 2 °C/min, 255–310 °C; flow rate: 0.5 mL/min; analysis time: 70 min; B: (column temperature) 5 min, 160°C; 3°C/min, 160–188°C; 1 min, 188 °C; 15 °C/min, 188–241 °C; 1 min, 241 °C; 2 °C/min, 241–282 °C; 5 °C/min, 282–310 °C; 5 min, 310 °C; flow rate: 1.0 mL/min; analysis time: 50 min	APCI-TOF-MS	Fruits of various olives species (*Olea* L.)	[170]
	Capillary column Supelco SPBM-5	two- and three-phase transfer catalysis (PTC), methyl iodide	carrier gas: He; injector temperature: 260 °C; detector temperature: 280 °C; furnace temperature: 5 min, 50 °C; 5 °C/min, 50–150 °C; 10 °C/min, 150–210 °C; analysis time: 45 min	MS	*Mentha spicata,* *Hypericum perforatum*	[179]
	non-polar RSL 200 BP	0.2 M trimethylaniline hydroxide (TMAH):H_2_O-free MeOH	carrier gas: N_2_; linear speed of the carrier 17.5 cm/s; 0–2 min, 280 °C and 235 °C; 1 °C/min, 280–290 °C; flow rate: 30 mL/min	FID	different samples form AFRC Institute of Plant Science Research and John Innes Institute, Norwich, U.K.	[167]
		Py:HMDS:TMCS				
	BPX5	Py:BSTFA:TMCS) (50:50:1 *v/v/v*)	carrier gas: He; injector temperature: 310 °C; 1 min, 100 °C; 30 °C/min, 100–210 °C; 2 °C/min, 210–240 °C; 4 °C/min, 240–270 °C; 5 °C/min, 270–310 °C; 5 min, 310 °C; flow rate: 1.5 mL/min	QMS	Propolis, *Chrysanthemum* sp, *Theobroma cacao* (bitter chocolate)	[171]
	Capillary column Low-bleed CP-Sil 8 CB-MS	TMCS (100 μL), BSTFA (200 μL) HMDS:TMCS:Py (3:1:9, *v/v/v*)	carrier gas: He; 2 °C/min, 70–135 °C; 10 min, 135 °C; 4 °C/min, 135–220 °C; 10 min, 220 °C; 3.5 °C/min, 220–270 °C; 20 min, 270 °C; injector temperature: 280 °C; detector temperature: 290 °C; flow rate: 1.9 mL/min	MS	*Teucrium polium*	[169]
	Quartz capillary column	Py:BSTFA (1:1 *v/v*)	carrier gas: He;injector temperature 220 °C; detector temperature: 270 °C; 2.3 °C/min 200–270 °C; 30 min, 270 °C	MS	*Aspalathus linearis*	[172]
Luteolin 7,3’,4’-trimethyl	Capillary column Permabond OV-1	---	carrier gas: N_2_, He; injector temperature: 300 °C; column temperature: 270 °C (isothermal); flow rate: 1.3 mL/min	FID	*Arnica alpina*	[168]
	Capillary column OV-1	---		MS		

Abbreviations: Py, pyridine, BSTFA, N,O-bis(trimethylsilyl)trifluoroacetamide; TMS, trimethylsily; HMDS, hexamethyldisilazane; TMCS, trimethylchlorosilane; APCI-TOF-MS, atmospheric pressure chemical ionization combined with quadrupole – time of flight mass spectrometry; FID, flame ionization detector; QMS, quadrupole mass spectrometry.

**Table 7:** Counter-current chromatography, as a preparative technique in the separation of luteolin derivatives.

Luteolin Derivative	Solvent System	Conditions	Detection	Analyzed Species	Ref.
Luteolin 6-glucoside	EtOAc:BuOH:H_2_O (2:1:3 *v/v/v*)	rotation speed: 800 rpm; flow rate (lower phase): 2.4 mL/min	UV-VIS, 254 nm; NMR; MS	*Patrinia villosa*	[173]
Luteolin 7-glucoside	EtOAc:EtOH:AcOH:H_2_O (4:1:0.25:5 *v/v/v/v*)	rotation speed: 800 rpm; flow rate (lower phase): 1.5 mL/min	UV, 254 nm; NMR; MS	*Paeonia suffruticosa*	[177]
Luteolin 6,8-dihexoside	Hx:EtOH:H_2_O (4:3:1 *v/v/v*)	rotation speed: 850 rpm; flow rate (lower phase): 2 mL/min	TLC; HPLC-UV-HRMS	*Lippia origanoides*	[178]
Luteolin 8-glucoside					
Luteolin 6-glucoside					
Luteolin 7-glucoside					

Abbreviations: AcOH, acetic acid; BuOH, butanol; EtOAc, ethyl acetate; EtOH, ethanol; HRMS, high resolution mass spectrometry; Hx, hexane, rpm, revolutions per minute.
